# Phylogenetic Relationship and Characterization of the Complete Mitochondrial Genome of the Cuckoo Species *Clamator coromandus* (Aves: Cuculidae)

**DOI:** 10.3390/ijms26030869

**Published:** 2025-01-21

**Authors:** Yu Zhang, Hao Gao, Fan Zhang, Chengxing Xia, Guopan Li, Shaobin Li

**Affiliations:** 1 College of Life Sciences, Yangtze University, Jingzhou 434025, China; 2 College of Fisheries, Huazhong Agricultural University, Wuhan 430070, China

**Keywords:** Cuculidae, genome organization, *Clamator coromandus*, mitochondrial genome, phylogenetic status

## Abstract

The chestnut-winged cuckoo (*Clamator coromandus*) is a bird species known for its brood parasitism, laying eggs in the nests of other bird species. However, there is a paucity of genetic information available for this species and their genus *Clamator*. In this study, we present the first complete mitochondrial genome sequence of *C*. *coromandus* and compare it with other species within the Cuculidae family. The mitogenome is a closed circular molecule consisting of 17,082 bp with an organization typical of the mitochondrial genomes of Cuculidae. Alignment of the control regions across Cuculidae species revealed substantial genetic variation and a significant abundance of AT content. A significant difference was detected in AT-skews between brood-parasitic and parental-care species. A distinctive long poly-C sequence was located at the 5′ end of domain I. Phylogenetically, *C*. *coromandus* is more closely related to *Piaya cayana* than *Ceuthmochares aereus*. The phylogenetic analysis indicated a general divergence between species with brood parasitism and those with parental care, with transitions between these behaviors within brood parasitism branches, suggesting multiple evolutionary occurrences of these traits. The complete mitogenome of *C*. *coromandus* serves as a valuable resource for further investigation into the taxonomic status and phylogenetic history of *Clamator* species.

## 1. Introduction

The family Cuculidae, the cuckoos, malkohas, ani’s, and roadrunners (147 species in 24 genera) [1], is notable for its diverse social behaviors and reproductive strategies. It is best known for its brood-parasitic members, which lay their eggs in the nests of other bird species, thereby relinquishing parental responsibilities [1,2]. Brood parasitism has evolved independently at least three times within this family, with many species exhibiting this behavior [3]. These cuckoos parasitize a remarkable variety of hosts globally, often laying eggs that closely mimic those of their hosts. Some species’ young may even eject host eggs from the nest upon hatching [4]. Primarily forest dwellers, these birds are often elusive, detected more by their distinctive, simple whistled calls than by sight.

The chestnut-winged cuckoo (*Clamator coromandus*), a member of the genus *Clamator*, is well-known for its striking plumage and unique behaviors [5]. The genus *Clamator* currently contains four species, but little information is available on them. The chestnut-winged cuckoo is a medium-sized bird, weighing between 66 and 86 g. It is notable for its metallic glossy black plumage, spiky crest, and narrow white nape-band. This species breeds across northern India and Nepal, extending eastward to southern and eastern China, with an altitudinal distribution up to 2450 m [6,7]. The chestnut-winged cuckoo typically lays its eggs in the nests of other bird species. As an insectivorous and migratory species, it winters in southern India, Sri Lanka, and the Greater Sundas. It thrives in diverse habitats, from wooded areas to scrub, bushes, orchards, plantations, and tall reedbeds [8].

However, *C*. *coromandus* remains a poorly understood species, with existing knowledge primarily limited to basic species accounts [1,4,5]. This study addresses this gap in research concerning its genetic characteristics by sequencing and reporting the complete mitochondrial genome of *C*. *coromandus*. We generated new data from this species and compared them with mitogenome sequences of other cuckoo species available in GenBank (Table 1). Our objectives were threefold: (a) to elucidate the sequences, features, and structures of the mitochondrial genome of *C*. *coromandus*, (b) to compare the mitogenome sequences between interspecific brood parasitism and parental care species, and (c) to describe the taxonomic status and phylogenetic relationships of *C*. *coromandus* within the family Cuculidae. The complete mitochondrial genome provides valuable insights into the evolutionary history and phylogenetic positioning of *C*. *coromandus* and related species within the broader avian phylogeny. Our findings contribute to a better understanding of species identification and genetic evolution within the Cuculidae family.

## 2. Results

### 2.1. Genome Content and Organization

**Figure 1:**
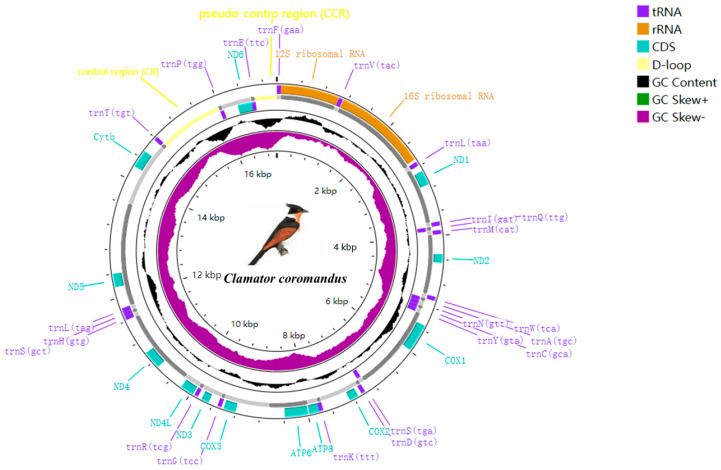
Complete mitochondrial genome organization and gene arrangement of *Clamator coromandus*. Genes coded on the H-strand are indicated in the outer ring, while the genes coded on the L-strand are indicated in the inner ring. Genes are abbreviated as follows: *ATP6* and *ATP8* (subunits 6 and 8 of ATPase), *COXI* − *COXIII* (cytochrome c oxidase subunits 1−3), *Cytb* (cytochrome b), and *ND1* − *ND6* and *ND4L* (NADH dehydrogenase subunits 1−6 and 4L). Ribosomal RNA of 12S and 16S, D-loop (control region), Trn, and one-letter amino acid abbreviations were used to label the corresponding tRNA genes.

The complete mitochondrial genome (mitogenome) of *C*. *coromandus* is a closed circular molecule and 17,082 bp in length. It contains the typical set of 37 genes found in vertebrate mitogenomes, including 13 protein-coding genes (PCGs), 22 transfer RNA (tRNA) genes, 2 ribosomal RNA (rRNA) genes (*12S rRNA* and *16S rRNA*), a control region (CR), and an additional pseudo-control region (CCR) (Table 2, Figure 1). Compared to the classical mitochondrial structure, the mitogenome of *C*. *coromandus* has four duplicate genes (*ND6*, *trnT*, *trnP*, and *trnE*) and an extra CR, referred to as a CCR. *ND6* and 8 tRNAs are transcribed from the light strand (L), while the other 12 PCGs, 14 tRNAs, 2 rRNAs, and 2 noncoding regions (CR, CCR) are located on the heavy (H) strand. The nucleotide composition of the mitogenome of *C*. *coromandus* is biased towards A and T (55.87%: A = 32.46%, T = 23.42%, G = 13.10%, and C = 31.03%). In the full mitogenome of *C*. *coromandus*, the AT- and GC-skews were 0.16 and −0.41. The A + T content of the CR was notably higher than that of the PCGs, tRNAs, and rRNAs (Table S1).

Mitochondrial genes overlapped by a total of 35 bp across 11 different locations, ranging from 1 to 10 bp. The longest overlap (10 bp) was found between the *ATP6* and *ATP8* genes, while the shortest overlap (1 bp) was observed in *12S rRNA*-*tRNA-Phe*, *tRNA-Val*-*12S rRNA*, *16S rRNA*-*tRNA-Val*, *tRNA-Leu*-*16S rRNA*, *tRNA-Met*-*tRNA-Gln*, *COXⅢ*-*ATP6*, and *tRNA-Leu*-*tRNA-Ser* genes. Additionally, some PCGsshared 1–10 nucleotides with adjacent tRNA genes. Furthermore, 17 intergenic spacers were present in the mitogenome of *C*. *coromandus*, totaling 86 bp. The longest spacer sequence was 15 nucleotides long, located between the *ND1* and *tRNA-Ile* genes (Table 2).

AT/GC skews were essentially similar in the mitochondrial PCGs, two rRNA genes, and the mitogenomes of other species of Cuculidae, except for a few genes (*ND1*, ND3, and *ND6*; Table S2). Notably, *Centropus bengalensis* exhibited a negative AT-skew in, while the other fourteen species had positive AT-skews (Table S2). For *ND3* genes, *Centropus unirufus* showed a negative AT-skew, whereas the other fourteen species had positive AT-skews. Apparent positive AT-skew and negative GC-skew biases were identified for all 12 PCGs (except for *ND1*, *ND3*, and *ND6*) encoded by the H-strand, whereas the reverse was detected in ND6 encoded by the L-strand (Table S2).

From the perspective of overall mitochondria and 13PCGs (Table S2), both brood parasites and parental care species exhibited similar AT/GC skews, with positive AT-skews and negative in GC-skews. Nonparametric tests (Table 3) revealed significant differences between brood parasites and parental care species in the AT-skews of overall mitochondria and 13PCGs (*p* < 0.05), while no significant differences were found in GC-skews (*p* > 0.05).

### 2.2. Protein-Coding Genes and Codon Usage Patterns

**Figure 2:**
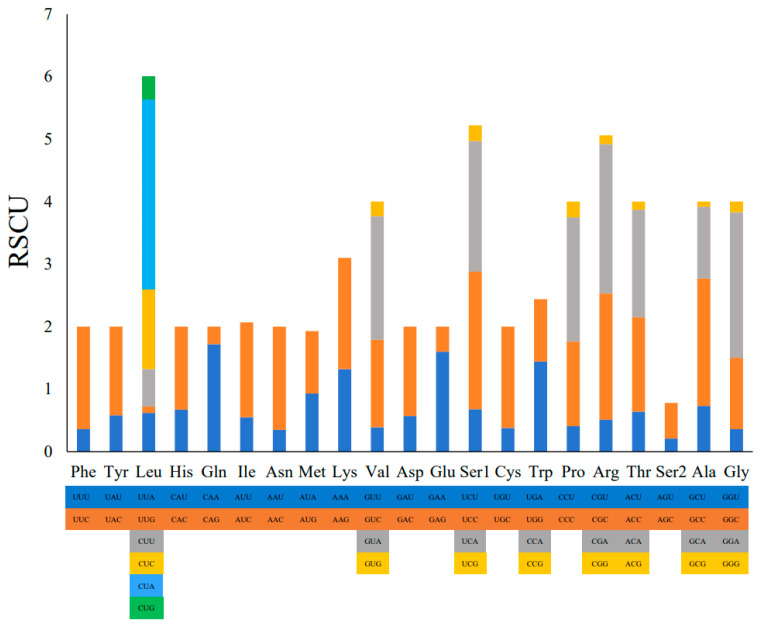
Relative Synonymous Codon Usage (RSCU) in the mitogenome of *C*. *coromandus*. The box below the bar indicates all codons encoding each amino acid, and the height of the above column indicates the sum of all RSCU values.

The 13 mitochondrial PCGs of *C*. *coromandus* span 11,391 bp, which account for 66.68% of the total mitogenome sequence. These PCGs consist of 3797 codons in total. Most PCGs use ATG as the start codon, except for *ND3* and *ND6*, which start with ATT and CTA, respectively (Table 2). TAA is the most frequent stop codon in the mitogenome of *C*. *coromandus. COXⅢ*, *ND4*, and *ND6* have incomplete stop codons (T-), *ND1* and *ND5* end with AGA, *ND2* ends with TAG, and *COXI* ends with AGG. All other stop codons are the standard terminal codon (TAA). Among the 64 available codons, the most commonly used ones were for proline (P; CCA; 3.92%), threonine (T; ACA; 3.95%), phenylalanine (F; UUC; 4.19%), isoleucine (I; AUC; 5.38%), and leucine (L; CUA; 7.31%). Leucine was the most frequently used amino acid in *C*. *coromandus* (Table 4, Figure 2). In contrast, the codons UUG, GUG, GCG, UGU, CGU, CGG, and GGG were rarely used, accounting for only 1.14% of the amino acids. Among the 60 codons analyzed, 28 had Relative Synonymous Codon Usage (RSCU) values greater than or equal to 1.00, indicating a preference for these codons. Leucine (Leu) has a higher preference for CUA, and the RSCU value is the highest in mitochondrial genome PCGs, which is 3.03. Methionine (AUG) and Tryptophan (UGG) showed no codon preference (RSCU = 1) (Table 4). The stop codons UAA, UAG, AGA, and AGG do not encode amino acids. Most amino acids have at least two different codons, while leucine has six codons (Figure 2). The base composition of the three codon positions of the 13 PCGs is shown in Table S3. The A + T content is 51.89% at the first position, 58.78% at the second position, and 54.54% at the third position. Hence, the second position has the highest A + T content, whereas the first position has the highest C + G content (48.11%; second position: 41.22%; third position: 45.46%). Moreover, the first positions and third positions have a positive AT-skew and negative GC-skews, but the second positions have negative AT- and GC-skews (Table S3). Among the 13 PCGs in the mitogenome of *C*. *coromandus* (Table 2), *ATP8* has the highest A + T content (58.9%), and *COXI* has the lowest (52.9%).

### 2.3. Transfer and Ribosomal RNA Genes

The mitogenome of *C*. *coromandus* contains the typical set of 22 tRNA genes with conventional secondary structures. The total length of mitochondrial tRNA genes is 1542 bp, with individual genes sizes ranging from 65 bp (*tRNA-Cys*) to 74 bp (*tRNA-Leu1* and *tRNA-Ser1*). Most tRNAs can be folded into the canonical cloverleaf secondary structure, except for *tRNA-Ser* (GCT), which lacks the DHU arm and loop but contains the TΨC arm and loop. A total of 31 unmatched base pairs were found in the tRNAs of *C*. *coromandus*, with the most common being G-U (22 instances), followed by U-U (2 instances), A-C (4 instances), U-C (1 instance), A-A (1 instance), and C-C (1 instance) (Figure S1). The *12S rRNA* gene is located between *tRNA-Phe* and *tRNA-Val*, while the *16S rRNA* gene is situated between *tRNA-Val* and *tRNA-Leu* (Figure 1). The lengths of the *12S rRNA* and *16S rRNA* were 975 bp and 1596 bp, respectively (Table 2). The base composition of the *12S rRNA* gene is 20.21% T, 26.97% C, 32.92% A, and 19.9% G, with an A + T content of 53.13%. The *16S rRNA* gene has a base composition of 21.74% T, 25.31% C, 34.71% A, and 18.23% G, with an A + T content of 56.45%.

### 2.4. Noncoding Regions

The noncoding region of the mitogenome of *C*. *coromandus* comprises two major control regions. The first CR is 1143 bp long and is located between *tRNA-Thr* and *tRNA-Pro*. The second is a short noncoding region, referred to as the CCR, which is 384 bp in length and situated after the *tRNA-Glu* gene (Figure 1). The CCR sequence is the shortest among all sequences in the mitogenome (Table 5). The base frequencies in the CR of *C*. *coromandus* are 30.53% T, 27.12% C, 29.31% A, and 13.04% G, whereas those in the CCR are 12.50% T, 31.25% C, 54.95% A, and 1.30% G.

**Figure 3:**
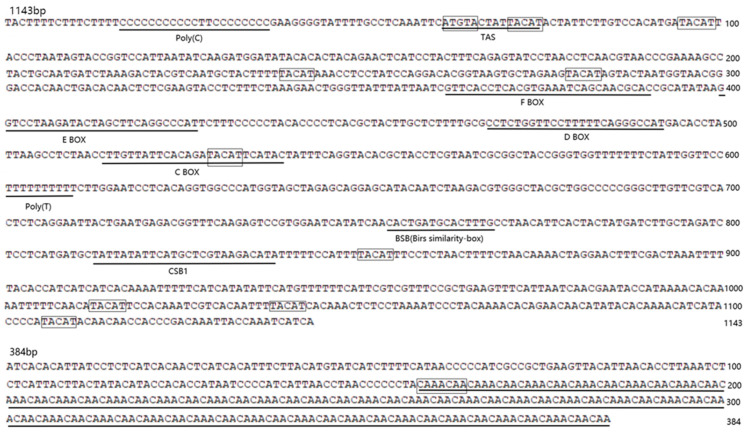
Nucleotide sequence forecast of the mitochondrial CR and CCR of *C. coromandus*. Several conserved motifs were identified along this fragment: putative ETAS elements (underlined), boxes (F, E, D, C), Bird similarity-box, blocks (CSB1), and repeated sequences (CAAACAA). Palindromic motifs (TACAT, ATGTA) are also represented in the figure. All complete repeating units are underlined for emphasis and easy identification.

**Figure 4:**
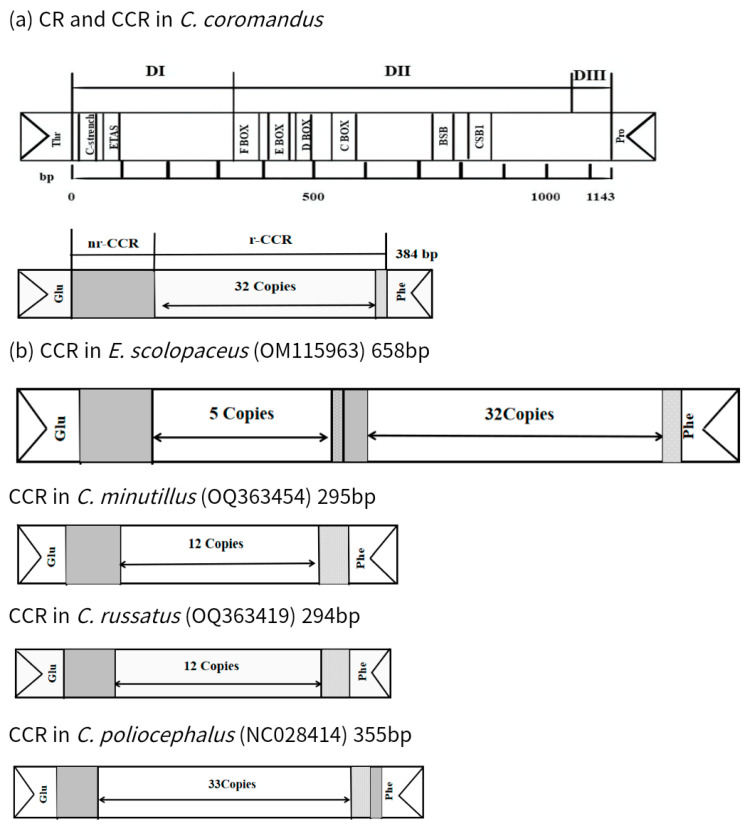
(**a**) Structure of CR and CCR in *C*. *coromandus*. Positions of the conserved motifs in CR and division into the three domains D I, D II, and D III are shown. (**b**) Structure of CCR in *Eudynamys scolopaceus*, *Chrysococcyx minutillus*, *Chrysococcyx russatus*, and *Cuculus poliocephalus*. nr-CCRs are depicted as gray bars. Regions containing incomplete units are depicted as hatched bars. nr-CCR, nonrepetitive region in CCR; r-CCR, repetitive region in CCR.

Hypervariable domain I (D I), located to the left (5′ end) of the CR, typically contains tandem repeat sequences with a copy number ranging from 1 to 8. The copy number varies both between species and among individuals within a species. At the 5′-end of D I, we observed a long continuous poly-C sequence (Figure 3), which appears to be a conserved feature. Conserved palindromic motifs 5′-TACAT-3′ and 5′-ATGTA-3′ were identified at the 5′ end of the CR of *C*. *coromandus* (Figure 3). The four conserved boxes (C, D, E, and F) and the CSB1 regions were also found in *C*. *coromandus* (Figure 3 and Figure 4a).

As shown in Figure 4a, the CCR of *C*. *coromandus* contains a 157 bp nonrepeating region (nr-CCR) at the 5′ end, followed by a cluster of tandem repeats at the 3′ end (r-CCR): 32 units of 7 bp repeat units (followed by a 6 bp incomplete repeat unit) (Figure 4, Table 5). In *Eudynamys scolopaceu*, two clusters of repetitive regions were observed in the CCR (Figure 4b): the first cluster (66 bp per unit, 5 times) at the 5′ region, and the second cluster (7 bp per unit, 23 times) at the 3′ region. These two regions are separated by a 28 bp nr-CCR and show no similarity to each other.

### 2.5. Phylogenetic Relationships

**Figure 5:**
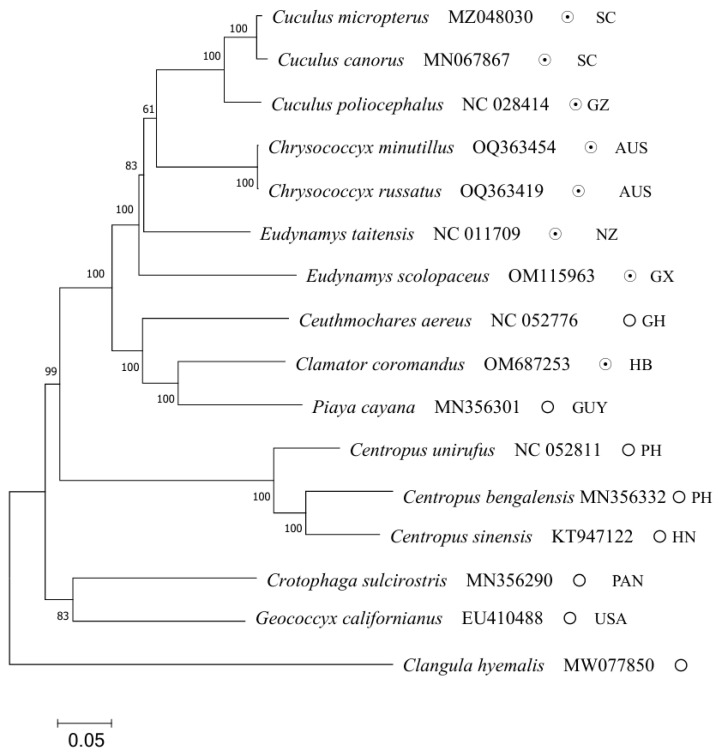
Results of phylogenetic analyses based on maximum likelihood (ML) analyses for 15 Cuculidae taxa based on 13 PCG sequences. *Clangula hyemalis* (MWO77850) was used as an outgroup. The ultrafast bootstrap values are shown at the nodes. ⊙ represents interspecific brood parasitism, ◯ represents parental care. SC: Sichuan Province of China; GZ: Guizhou Province of China; AUS: Australia; NZ: New Zealand; GX: Guangxi Province of China; GH: Ghana; HB: Hubei Province of China; GUY: Guyana; PH: Philippines; HN: Hainan Province of China; PAN: Panama; USA: United States of America.

**Figure 6:**
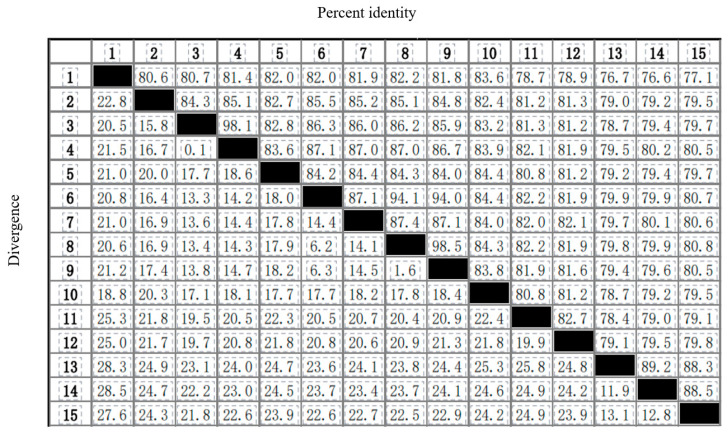
Nucleotide sequence identity alignment of 13 PCGs in 15 species of Cuculidae. The numbers in the 1st ranks and rows of this figure represent the species numbers in Table 1.

**Figure 7:**
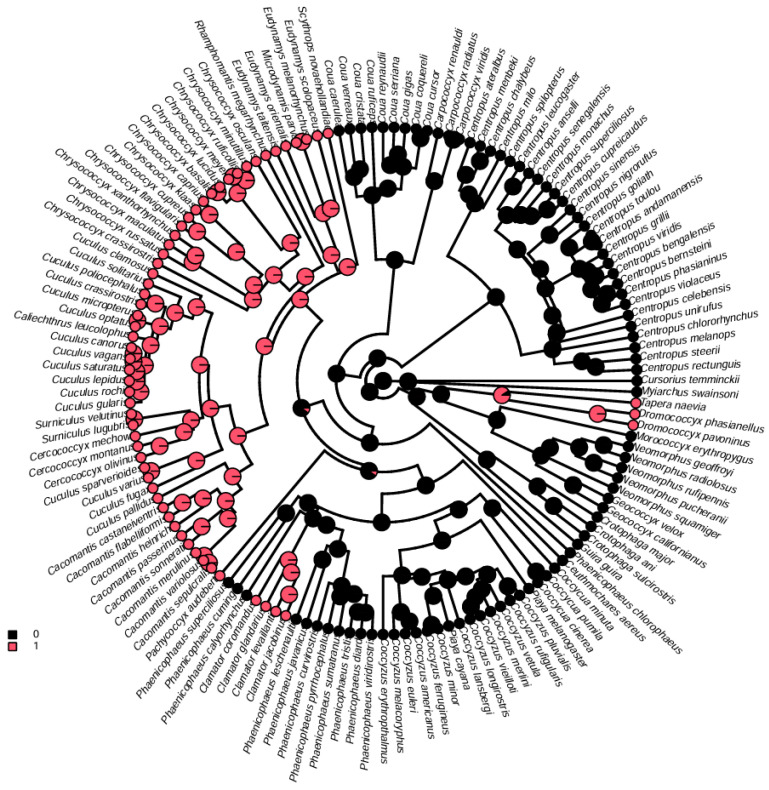
Ancestral trait reconstruction of Cuculidae: 0 represents parental care, while 1 signifies brood parasitism. *Myiarchus swainsoni* and *Cursorius temminckii* were utilized as outgroups.

To explore the molecular phylogenetics and evolution of Cuculidae, a phylogenetic analysis was conducted based on the nucleotide sequence data of the 13 PCGs of *C*. *coromandus* and 14 other species ofCuculidae. *Clangula hyemalis* was used as the outgroup. The maximum likelihood tree is shown in Figure 5. The maximum likelihood trees showed stable topological structures with high nodal support values. Based on 14 other complete mitogenome sequences retrieved from NCBI GenBank (Table S1), most internal nodes were well-supported by bootstrap values (Figure 5). The results indicate that the genus *Eudynamys* is not monophyletic because *Eudynamys taitensis* was more closely related to species of *Cuculus* and *Chrysococcyx* than to *E. scolopaceus* (Figure 5). The phylogenetic tree reveals that among the 14 other species of Cuculidae, *C*. *coromandus* was closely related to *Ceuthmochares aereus* and *Piaya cayana*, with *C*. *coromandus* being most closely related to *P. cayana*. Comparisons of genetic divergence also indicated that *C*. *coromandus* was most similar *P. cayana* (Figure 6). The phylogenetic tree also indicates a general separation between interspecific brood parasitism and parental care, with a transition towards brood parasitism occurring twice within the evolutionary history of the cuckoos, once in the lineage leading to *C. coromandus* and once in the lineage leading to the clade formed by *Eudynamys*, *Cuculus,* and *Chrysococcyx*. We have marked the locations of the sampling points on the phylogenetic tree and found that all sampling sites of species in the *Cuculus* genus are located in China, with all species within this genus being brood parasites (Figure 5). From a phylogenetic perspective, cuckoos, as migratory birds, are distributed across various regions worldwide. We reconstructed the ancestral characteristics of the Cuculidae family (Figure 7). The phylogenetic analysis indicates a transition from parental care to brood parasitism within the cuckoo lineage. The results of our ancestral state reconstruction align with those presented in Figure 5.

## 3. Discussion

The complete mitochondrial genome of *C*. *coromandus* is a closed circular molecule, which is similar to other members of the Cuculidae family. It contains the typical set of 37 genes found in vertebrate mitogenomes. This configuration is consistent with other Cuculidae species [9]. Previous studies have suggested that the extra CR could confer selective advantages, such as a replicative advantage, to duplicated mitochondrial DNAs over those with a single CR [10,11]. In addition, we observed mitochondrial genome rearrangements in *C*. *coromandus*. Specifically, an additional noncoding sequence was found between the *tRNA-Glu* and *tRNA-Phe* genes aside from the original control region located between *tRNA-Thr* and *tRNA-Pro* [12]. The presence of the CCR is noted in the mitochondrial genomes of numerous bird species [13]. Other Cuculiformes species also possess a CR and a short CCR [14,15]. Similar rearrangements have been detected in representatives of Passeriformes, Procellariiformes, Falconiformes, Piciformes, and Psittaciformes [16,17,18]. Studies have also found that the duplication of CRs in the mitochondrial genome is associated with longer lifespans in birds compared to mammals of similar weight [19]. The gene arrangement pattern of the mitochondrial genome of *C*. *coromandus* is identical to that of other species of Cuculidae, all of which have the remnant CCR gene order, differing from the standard bird gene order [20,21]. AT-skew, GC-skew, and A + T content are commonly used to assess differences in the nucleotide composition of mitochondrial genomes [22]. This mitogenome has greater A + T content (55.87%) (Table S1) compared to the G + C content (44.13%), which is similar to other species of Cuculidae [23,24]. Some PCGsshare nucleotides with adjacent tRNA genes. This compact and economical arrangement is a common feature of mitochondrial DNA [25].

*COXⅢ*, *ND4*, and *ND6* have incomplete stop codons (T-). These incomplete stop codons are presumably completed through post-transcriptional polyadenylation using a poly-A tail [26]. Codon preference refers to the phenomenon where specific codons are utilized more prevalently than others in the DNA or RNA sequences of certain organisms, which can influence gene expression and protein assembly [27]. Leucine (Leu) has a higher preference for CUA. Methionine (AUG) and Tryptophan (UGG) showed no codon preference. Consistent with other vertebrates [28], there is a strong bias against G at the third codon position in all 13 PCGs. The G content is 5.93% at the third codon position (Table S2).

Meanwhile, *tRNA-Ser* (GCT) lacks a DHU arm and loop. This feature is typical of vertebrate mitogenomes [29] and is generally observed in vertebrate tRNA genes [30]. Structural compensation mechanisms may render the missing arm in *tRNA-Ser* functional [31]. Most anticodons are identical to those observed in other Cuculidae species, and the CCA 3′-terminal group is added during processing. A total of 31 unmatched base pairs were found in the tRNAs of *C*. *coromandus*. Stem mismatches are common in tRNA genes and are likely repaired through post-transcriptional editing [32]. Compared to other birds, most mismatched nucleotides were G–U pairs, which can form weak bonds in tRNAs and noncanonical pairs in tRNA secondary structures [33]. The gene order, arrangement, length, base composition, and RNA structure of the ribosomal RNA genes (*12S rRNA* and *16S rRNA*) are similar to those in other Cuculidae birds.

Both vertebrates and invertebrates CRs exhibit high A + T content and possess replication initiation features [34]. The A + T content of the CR of *C*. *coromandus* is 59.8%, and the CCR is 67.5% (Table 2). The control region of *C*. *coromandus* shares structural similarities with *Chrysococcyx minutillus* and *Chrysococcyx russatus*, and it exhibits common characteristics found in other birds [35]. Generally, three distinct CR domains are recognized: (a) the highly variable, left-end domain I (D I); (b) the conserved, central domain (D II); and (c) the right-end domain III (D III) [36]. A conserved palindromic motif can be identified at the 5′ end of the CR of *C*. *coromandus* [37]. The CCR of *C*. *coromandus* contains a cluster of tandem repeats (Figure 4, Table 5). In *E. scolopaceu*, two clusters of repetitive regions were observed in the CCR (Figure 4b). These variable tandem repeats are the primary cause of length variability in mitochondrial genome control regions and the entire mitogenome [38,39].

In this study, a phylogenetic analysis was conducted on the nucleotide sequence data of 13 PCGs from *C. coromandus* and 14 otherspecies of Cuculidae. *C. hyemalis* was selected as the outgroup. The complete mitochondrial genome of *C*. *coromandus* will hopefully contribute to the systematic and molecular identification of Cuculidae. The phylogenetic trees indicate that *E. scolopaceus* is not a sister-taxon of *E. taitensis*. Our current study provides further evidence for a closer phylogenetic relationship between *C. coromandus* and *P. cayana* compared to other species included in our data [40], suggesting the need for further investigation. *C. coromandus*, as a brood-parasitic species, is more closely related to parental care (*P. cayana*) (Figure 5). Species that are more similar in terms of brood parasitic behavior are not necessarily more closely related in the phylogeny of the Cuculidae family [41]. This result is consistent with our study. All clades were well-resolved, illustrating that despite their rapid evolutionary rates, mitogenomes possess species-specific evolutionary relationships that can be effectively elucidated through enhanced taxon sampling [42]. By adding sampling points for species on the phylogenetic tree, we observed that cuckoos inhabit numerous regions globally; however, all sampling points for species within the *Cuculus* genus are confined to China. Research into the migration routes and wintering grounds of *Cuculus canorus* has demonstrated that their migration paths exhibit relative stability with minimal variation, ultimately leading them to winter in Africa [43,44]. From the perspective of the phylogenetic tree, the sampling point for *C. canorus* was situated in Sichuan Province, China, thereby providing valuable data for studying the migratory behavior of this species. Darwin initially proposed that parasitic cuckoos evolved from parental cuckoos [45]. Our findings demonstrate a general distinction between interspecific brood parasitism and parental care, with transitions between these behaviors occurring at least twice within the evolutionary history of cuckoos. Following our reconstruction of ancestral traits within Cuculidae (Figure 7), we found that these results corroborate findings from this study, which supports the hypothesis regarding a transition from parental care to interspecific brood parasitism.

## 4. Materials and Methods

### 4.1. Sample Collection and Genomic DNA Extraction

**Figure 8:**
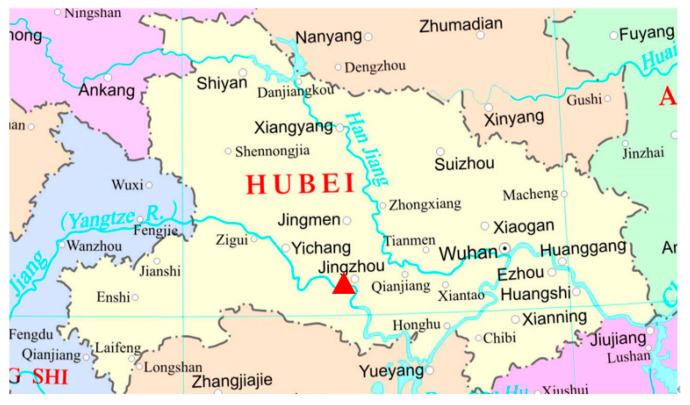
Sampling location map of the chestnut-winged cuckoo, *C*. *coromandus* (red triangle represents the sampling point near the east gate of the ancient city, Jingzhou County, Hubei Province of China).

Muscle tissue from a male *C*. *coromandus* (Figure S2) was collected from an individual that died accidentally from glass collision near the east gate of the ancient city in Jingzhou County, Hubei Province, China (latitude: 30°20′5″ N; longitude: 112°12′57″ E; Figure 8) on 29 October 2022. This species can be easily distinguished by its body size and plumage patterns [46]. The sex of the individuals was determined using a pair of universal primers for avian sex identification [47]. Whole-genome DNA was extracted from the muscle tissue according to the protocol of TIANamp Genomic DNA kits (Tiangen, Beijing, China). The remaining sample is now stored in herbarium room 317 of the #1 Teaching Building on the West campus of Yangtze University (www.yangtzeu.edu.cn, Dr. Shaobin Li, shaobinlee@126.com).

### 4.2. Mitochondrial DNA Amplification and Sequencing

High-throughput sequencing technology was employed to sequence the mitochondrial genome of *C*. *coromandus* from the muscle tissue. Genomic DNA was extracted, and a DNA library was constructed using the TruSeq Nano DNA HT Sample Prep Kit (Illumina, San Diego, CA, USA). The library was sequenced on an Illumina HiSeq/NovaSeq platform with a 2 × 150 paired-end sequencing strategy.

### 4.3. Assembly, Annotation, and Analysis of the Mitochondrial Genome

The complete mitochondrial genome sequence of *C*. *coromandus* was assembled using the SeqMan module of DNASTAR software (DNASTAR, Inc., Madison, WI, USA) [48]. PCGs were identified through sequence comparisons with known sequences of other birds using the CLUSTAL W program. Transfer RNA (tRNA) genes and their secondary structures were identified using tRNAscan-SE 1.21 [49] or based on their proposed secondary structures and anticodons [50]. Ribosomal RNA genes (rRNAs) were identified via NCBI BLAST search and comparisons. An online tool, CGVIEW, was employed to draw the mitogenome structure map (https://cgview.ca/ accessed on 17 January 2025) [51]. Codon usage and nucleotide composition statistics were computed using MEGA 7.0 [52]. If RSCU = 1, there is no preference for the use of this codon, and if RSCU > 1, the codon is used preferentially by amino acids, while if RSCU < 1, the codon usage is contrary. Composition skew analysis was performed with the formulas AT-skew = [A − T]/[A + T] and GC-skew = [G − C]/[G + C] [53]. The 15 species of Cuculidae were divided into two categories, namely, brood parasites and parental care (Table 1). We employed the non-parametric Mann–Whitney U test to determine whether AT/GC-skew nucleotides differ between brood parasites and parental care using SPSS 20.0 software. Because the sample size was too small (brood parasites: n = 8; parental care: n = 7) and the two groups of data did not follow a normal distribution, non-parametric tests were used for comparison. Based on the next-generation sequencing method, the complete sequence of the mitochondrial genome of *C*. *coromandus* was assembled and compared with the species of the same family reported in GenBank to determine the start and end points of the control region. By conducting a comprehensive alignment of the reported mitochondrial control region structures in birds [35,54,55,56], we analyzed the structure of the mitochondrial control region of *C. coromandus* and identified three regions within its control region: the termination-associated sequence (ETAS), the central conserved region (CD), and the conserved sequence block (CSB). The core of ETAS is TACAT, and its reverse complementary sequence is ATGTA, which can form a hairpin structure. In the CD, three characteristic fragments were identified, namely CSB-D, CSB-E, and CSB-F. The CSB typically contains three conserved sequences: CSB1, CSB2, and CSB3. The sequence of the complete mitogenome of *C*. *coromandus* was deposited in GenBank under accession number OM687253.

### 4.4. Phylogenetic Analysis

The PCGs were extracted from mitogenomes using PhyloSuite v.1.1.16 [57]. Repetitive sequences were removed to obtain a final set of protein-coding regions for constructing the phylogenetic tree. Sequence data were initially aligned using ClustalX 1.83 [58] with default parameters. The mitogenome of *C*. *coromandus* was analyzed in this study, along with 14 other Cuculidae mitogenomes retrieved from NCBI GenBank (Table S1). The concatenated sequences of the 13 PCGs of complete mitochondrial genomes were used. The best-fit substitution models (GTR  +  G  +  I) of nucleotide sequences were tested using MEGA. We evaluated the confidence of each branch by performing 1000 bootstrap replications. Finally, the trimmed PCGs were concatenated to construct a phylogenetic tree using MEGA. We added the sampling points of species samples on the phylogenetic tree. With the exception of the sampling site for *C. coromandus*, all other sampling locations for 14 additional cuckoo species were obtained from previous studies [14,15,23,24,59,60]. We download 1000 alternative phylogenetic trees of cuckoos from birdtree.org [61] and constructed a maximum clade credibility tree, which is adequate for addressing phylogenetic uncertainty [62]. The parasitic behavior state of ancestral nodes is considered the response variable, while the parasitic behavior of extant cuckoo species (i.e., non-brood parasitic cuckoos or brood parasitic cuckoos) is considered the independent variable (n = 142 species). We reconstructed ancestral states of brood-parasitism’s presence using the “ace” Function in R package, and our model choice was “ER” (equal-rates model), with the type being discrete character [63]. All 142 species of cuckoos included in the analysis are present in previous studies [3,4,6,41,64]. All analyses were carried out in R 4.3.3 [65] using the R package phytools [66].

## 5. Conclusions

This study represents the first comprehensive report and analysis of the complete mitochondrial genome of *C*. *coromandus*. The mitogenome structure of *C*. *coromandus* is typical for birds and shows high similarity to other reported Cuculidae mitogenomes. Our results revealed a significant difference in AT-skews between interspecific brood parasitism and parental care. Notably, a long continuous poly-C sequence was discovered at the 5′ end of the displacement loop (D-loop) region. Additionally, one type of tandem repeat unit was identified in the CCR. Phylogenetic analyses indicate a closer relationship between *C*. *coromandus* and *P. cayana* compared to *C*. *coromandus* and *C. aereus*. While interspecific brood parasitism and parental care are generally separated, transitions between these behaviors occur occasionally within the evolutionary tree. This suggests that both traits have evolved multiple times across different evolutionary lineages. Consequently, our findings provide a valuable resource for future studies on the mitochondrial genomic evolution of the Cuculidae family. However, many aspects of Cuculidae phylogeny remain unresolved. Further analysis using additional molecular markers, such as nuclear genes or the genome, and larger taxon sampling is necessary to clarify the phylogenetic relationships among species within this family.

## Figures and Tables

**Table 1:** Mitogenomes of Cuculidae with accession numbers used in this study.

Species Number	Scientific Name	GenBank Number	Reproductive Strategy
1	*Clamator coromandus*	OM687253	interspecific brood parasitism
2	*Eudynamys scolopaceus*	OM115963	interspecific brood parasitism
3	*Chrysococcyx minutillus*	OQ363454	interspecific brood parasitism
4	*Chrysococcyx russatus*	OQ363419	interspecific brood parasitism
5	*Ceuthmochares aereus*	NC052776	parental care
6	*Cuculus poliocephalus*	NC028414	interspecific brood parasitism
7	*Eudynamys taitensis*	NC011709	interspecific brood parasitism
8	*Cuculus micropterus*	MZ048030	interspecific brood parasitism
9	*Cuculus canorus*	MN067867	interspecific brood parasitism
10	*Piaya cayana*	MN356301	parental care
11	*Crotophaga sulcirostris* *	MN356290	parental care
12	*Geococcyx californianus*	EU410488	parental care
13	*Centropus bengalensis*	MN356332	parental care
14	*Centropus sinensis*	KT947122	parental care
15	*Centropus unirufus*	NC052811	parental care

*: multiple females lay in a joint nest.

**Table 2:** Characteristics of the mitochondrial genome of *C. coromandus*.

Gene	Position		Sizes	Codon		Intergenic Nucleotide ^b^	Strand ^c^	A + T%
	From	To	Nucleotide (bp)	Start	Stop ^a^			
*tRNA-Phe*	1	70	70				H	45.7
*12S rRNA*	70	1044	975			−1	H	53.1
*tRNA-Val*	1044	1115	72			−1	H	58.3
*16S rRNA*	1115	2710	1596			−1	H	56.5
*tRNA-Leu*	2710	2783	74			−1	H	48.7
*ND1*	2795	3772	978	ATG	AGA	11	H	54.8
*tRNA-Ile*	3788	3858	71			15	H	57.8
*tRNA-Gln*	3871	3941	71			12	L	67.6
*tRNA-Met*	3941	4009	69			−1	H	53.6
*ND2*	4010	5050	1041	ATG	TAG	0	H	57.5
*tRNA-Trp*	5049	5118	70			−2	H	62.9
*tRNA-Ala*	5120	5188	69			1	L	56.5
*tRNA-Asn*	5192	5264	73			3	L	46.6
*tRNA-Cys*	5269	5333	65			4	L	56.9
*tRNA-Tyr*	5334	5403	70			0	L	52.9
*COXⅠ*	5405	6955	1551	ATG	AGG	1	H	52.9
*tRNA-Ser*	6947	7020	74			−9	L	54.1
*tRNA-Asp*	7023	7091	69			2	H	59.4
*COXⅡ*	7093	7776	684	ATG	TAA	1	H	53.1
*tRNA-Lys*	7781	7848	68			4	H	60.3
*ATP8*	7850	8017	168	ATG	TAA	1	H	58.9
*ATP6*	8008	8691	684	ATG	TAA	−10	H	54.8
*COXⅢ*	8691	9474	784	ATG	T-	−1	H	54.2
*tRNA-Gly*	9475	9543	69			0	H	62.3
*ND3*	9544	9895	352	ATT	TAA	0	H	56.0
*tRNA-Arg*	9900	9968	69			4	H	65.2
*ND4L*	9970	10,266	297	ATG	TAA	1	H	53.2
*ND4*	10,260	11,637	1378	ATG	T-	−7	H	55.5
*tRNA-His*	11,638	11,707	70			0	H	65.7
*tRNA-Ser*	11,708	11,773	66			0	H	51.5
*tRNA-Leu*	11,773	11,843	71			−1	H	59.2
*ND5*	11,844	13,652	1809	ATG	AGA	0	H	56.7
*Cytb*	13,661	14,803	1143	ATG	TAA	8	H	55.0
*tRNA-Thr*	14,807	14,876	70			3	H	62.9
*Control region*	14,877	16,019	1143			0	H	59.8
*tRNA-Pro*	16,020	16,089	70			0	L	64.3
*ND6*	16,101	16,622	522	CTA	T-	11	L	54.0
*tRNA-Glu*	16,627	16,698	72			4	L	56.9
*Pseudo-control region*	16,699	17,082	384			0	H	67.5

^a^ T—represents incomplete stop codons. ^b^ Intergenic bp indicates gap nucleotides (positive value) or overlapped nucleotides (negative value) between two adjacent genes. ^c^ H and L indicate genes transcribed on the heavy and light strands, respectively.

**Table 3:** Results of non-parametric Mann–Whitney test analysis of AT/GC-skews in 13 PCGs and overall.

			M (*Q_L_*, *Q_U_*)	*Z*	*P*
13PCGs	AT-skews	interspecific brood parasitism	0.100 (0.090~0.123)	−2.639	0.008
		parental care	0.070 (0.050~0.080)		
	GC-skews	interspecific brood parasitism	−0.405 (−0.425~−0.400)	−0.945	0.344
		parental care	−0.400 (−0.420~−0.370)		
overall	AT-skews	interspecific brood parasitism	0.160 (0.150~0.175)	−2.598	0.009
		parental care	0.140 (0.110~0.140)		
	GC-skews	interspecific brood parasitism	−0.400 (−0.410~−0.393)	−1.413	0.158
		parental care	−0.390 (−0.410~−0.370)		

Note: M stands for median, *Q_L_* stands for lower quartile, and *Q_U_* stands for upper quartile.

**Table 4:** Codon usage in the mitochondrial protein-coding genes of *C*. *coromandus*.

Codon	Count	RSCU	%	Codon	Count	RSCU	%	Codon	Count	RSCU	%	Codon	Count	RSCU	%
UUU (F)	34.0	0.36	0.92	UCU (S)	36.0	0.68	0.97	UAU (Y)	44.0	0.58	1.19	UGU (C)	5.0	0.38	0.14
UUC (F)	155.0	1.64	4.19	UCC (S)	116.0	2.20	3.14	UAC (Y)	107.0	1.42	2.90	UGC (C)	21.0	1.62	0.57
UUA (L)	55.0	0.62	1.49	UCA (S)	110.0	2.09	2.98	UAA (*)	56.0	0.99	1.52	UGA (W)	81.0	1.44	2.19
UUG (L)	9.0	0.10	0.24	UCG (S)	13.0	0.25	0.35	UAG (*)	32.0	0.57	0.87	UGG (W)	11.0	1.00	0.30
CUU (L)	53.0	0.60	1.43	CCU (P)	30.0	0.41	0.81	CAU (H)	52.0	0.67	1.41	CGU (R)	7.0	0.51	0.19
CUC (L)	114.0	1.28	3.08	CCC (P)	98.0	1.35	2.65	CAC (H)	103.0	1.33	2.79	CGC (R)	28.0	2.02	0.76
CUA (L)	270.0	3.03	7.31	CCA (P)	145.0	1.99	3.92	CAA (Q)	119.0	1.72	3.22	CGA (R)	33.0	2.39	0.89
CUG (L)	33.0	0.37	0.89	CCG (P)	18.0	0.25	0.49	CAG (Q)	19.0	0.28	0.51	CGG (R)	2.0	0.14	0.05
AUU (I)	72.0	0.55	1.95	ACU (T)	54.0	0.64	1.46	AAU (N)	29.0	0.35	0.78	AGU (S)	11.0	0.21	0.30
AUC (I)	199.0	1.52	5.38	ACC (T)	128.0	1.51	3.46	AAC (N)	135.0	1.65	3.65	AGC (S)	30.0	0.57	0.81
AUA (M)	122.0	0.93	3.30	ACA (T)	146.0	1.72	3.95	AAA (K)	99.0	1.78	2.68	AGA (*)	10.0	0.72	0.27
AUG (M)	24.0	1.00	0.65	ACG (T)	11.0	0.13	0.30	AAG (K)	12.0	0.22	0.32	AGG (*)	3.0	0.22	0.08
GUU (V)	12.0	0.39	0.32	GCU (A)	46.0	0.73	1.24	GAU (D)	19.0	0.57	0.51	GGU (G)	15.0	0.36	0.41
GUC (V)	43.0	1.40	1.16	GCC (A)	129.0	2.04	3.49	GAC (D)	48.0	1.43	1.30	GGC (G)	47.0	1.14	1.27
GUA (V)	61.0	1.98	1.65	GCA (A)	73.0	1.15	1.98	GAA (E)	76.0	1.60	2.06	GGA (G)	96.0	2.33	2.60
GUG (V)	7.0	0.23	0.19	GCG (A)	5.0	0.08	0.14	GAG (E)	19.0	0.40	0.51	GGG (G)	7.0	0.17	0.19

Note: The asterisk (*) represents the stop codon.

**Table 5:** Sequence characteristics of CCRs in 15 species of Cuculidae birds.

Scientific Name	Accession Number	Genome Length (bp)	CCR		
			Types	Tandem Repeats	Single Repeat Unit
*C. coromandus*	OM687253	17,082	1	32.6 × (7)	CAAACAA
*E. scolopaceus*	OM115963	17,610	2	5.1 × (66)	CACCACCACCCTCCCCGCTGAAATTACATTAACAAATTACATCATATCACCCATAATTTTATATTT
				23.1 × (7)	AAACAAC
*C. minutillus*	OQ363454	17,190	1	12.8 × (12)	AAAACAAACAAC
*C. russatus*	OQ363419	17,211	1	12.8 × (12)	AAAACAAACAAC
*C. aereus*	NC052776	17,187	-	-	-
*C. poliocephalus*	NC028414	17,508	1	33.4 × (7)	CAACAAA
*E. taitensis*	NC011709	17,559	1	16.9 × (7)	AAACAAC
*C. micropterus*	MZ048030	17,541	-	-	-
*C. canorus*	MN067867	17,457	-	-	-
*P. cayana*	MN356301	17,007	-	-	-
*C. sulcirostris*	MN356290	16,933	-	-	-
*G. californianus*	EU410488	17,091	1	31.7 × (7)	CAAACAA
*C. bengalensis*	MN356332	17,107	-	-	-
*C. sinensis*	KT947122	17,159	-	-	-
*C. unirufus*	NC052811	17,089	-	-	-

Note: The bars indicate that the species have no CCR. In a complete mitochondrion sequence, there are tandem repeat units outside of the CCR region, but this study (Table 5) only counted the tandem repeat units in the CCR segment. 32.6 × (7) = 32.6 repeats of 7 bp (a single repeat unit is 7 bp in length).

## Data Availability

The complete mitogenome of *C*. *coromandus* was deposited in GenBank under the accession number OM687253. Raw sequence data are deposited in the NCBI Sequence Read Archive under accession number SRR18063420.

## Supplementary Materials

The following supporting information can be downloaded at: https://www.mdpi.com/article/10.3390/ijms26030869/s1.
