# Supplementary material for: Phylogenetic Relationship and Characterization of the Complete Mitochondrial Genome of the Cuckoo Species Clamator coromandus (Aves: Cuculidae)
Source: Int J Mol Sci. 2025 Jan 21;26(3):869. doi: 10.3390/ijms26030869 (PMC11816960; doi:10.3390/ijms26030869)
Supplement: Supplementary file 1 [file ijms-26-00869-s001.zip › ijms-3325532-supplementary.pdf]

**Table S1** Comparison of whole genome sizes and A + T contents, together with GenBank accession numbers of the mitogenomes of 15 Cuculidae birds.

| Species | Accession no. | Total | | | | 13 PCGs | | | | 16S rRNA gene | | | | 12S rRNA gene | | | | 22 tRNA | | | | CR | | | |
|---|---|---|---|---|---|---|---|---|---|---|---|---|---|---|---|---|---|---|---|---|---|---|---|---|---|
| | | Length (bp) | A+T (%) | AT skew | GC skew | Length (bp) | A+T (%) | AT skew | GC skew | Length (bp) | A+T (%) | AT skew | GC skew | Length (bp) | A+T (%) | AT skew | GC skew | Length (bp) | A+T (%) | AT skew | GC skew | Length (bp) | A+T (%) | AT skew | GC skew |
| *Clamator coromandus* | OM687253 | 17082 | 55.87 | 0.16 | -0.41 | 11388 | 55.07 | 0.15 | -0.48 | 1596 | 56.45 | 0.23 | -0.16 | 975 | 53.13 | 0.24 | -0.15 | 1542 | 57.65 | 0.15 | -0.19 | 1527 | 61.76 | 0.16 | -0.47 |
| *Eudynamys scolopaceus* | OM115963 | 17610 | 57.58 | 0.18 | -0.42 | 11400 | 56.78 | 0.13 | -0.42 | 1593 | 56.56 | 0.25 | -0.17 | 972 | 54.42 | 0.21 | -0.15 | 1548 | 57.82 | 0.17 | -0.21 | 1203 | 62.09 | 0.11 | -0.53 |
| *Chrysococcyx minutillus* | OQ363454 | 17190 | 55.64 | 0.18 | -0.41 | 11388 | 55.12 | 0.09 | -0.40 | 1590 | 54.84 | 0.22 | -0.17 | 974 | 55.03 | 0.19 | -0.15 | 1550 | 58.13 | 0.15 | -0.17 | 1635 | 57.98 | 0.13 | -0.40 |
| *Chrysococcyx russatus* | OQ363419 | 17211 | 56.75 | 0.15 | -0.39 | 11391 | 56.19 | 0.09 | -0.41 | 1590 | 56.98 | 0.23 | -0.16 | 974 | 55.24 | 0.20 | -0.15 | 1549 | 58.68 | 0.15 | -0.18 | 1657 | 59.44 | 0.12 | -0.43 |
| *Ceuthmochares aereus* | NC052776 | 17187 | 57.04 | 0.14 | -0.39 | 11394 | 56.72 | 0.08 | -0.40 | 1592 | 56.78 | 0.22 | -0.17 | 975 | 54.67 | 0.16 | -0.14 | 1546 | 58.28 | 0.16 | -0.17 | 1134 | 58.47 | -0.05 | -0.30 |
| *Cuculus poliocephalus* | NC028414 | 17508 | 57.48 | 0.16 | -0.40 | 11391 | 56.65 | 0.10 | -0.41 | 1599 | 57.47 | 0.22 | -0.17 | 972 | 55.35 | 0.20 | -0.15 | 1545 | 58.45 | 0.15 | -0.19 | 1945 | 62.78 | 0.16 | -0.49 |
| *Eudynamys taitensis* | NC011709 | 17559 | 56.64 | 0.14 | -0.39 | 11394 | 56.01 | 0.08 | -0.40 | 1598 | 57.01 | 0.21 | -0.16 | 967 | 54.91 | 0.18 | -0.14 | 1542 | 57.59 | 0.16 | -0.18 | / | / | / | / |
| *Cuculus micropterus* | MZ048030 | 17541 | 57.36 | 0.16 | -0.40 | 11393 | 56.6 | 0.10 | -0.40 | 1599 | 57.16 | 0.22 | -0.16 | 972 | 55.04 | 0.21 | -0.16 | 1538 | 58.39 | 0.14 | -0.19 | 1650 | 62.55 | 0.00 | -0.40 |
| *Cuculus canorus* | MN067867 | 17457 | 57.32 | 0.15 | -0.40 | 11391 | 56.81 | 0.10 | -0.40 | 1599 | 57.29 | 0.21 | -0.16 | 972 | 55.25 | 0.21 | -0.15 | 1545 | 57.99 | 0.17 | -0.18 | 1266 | 60.74 | 0.13 | -0.47 |
| *Piaya cayana* | MN356301 | 17007 | 57.18 | 0.14 | -0.39 | 11403 | 56.42 | 0.08 | -0.41 | 1604 | 58.67 | 0.19 | -0.13 | 973 | 54.47 | 0.21 | -0.15 | 1541 | 59.51 | 0.15 | -0.17 | 1139 | 59.44 | -0.02 | -0.35 |
| *Crotophaga sulcirostris* | MN356290 | 16933 | 55.81 | 0.14 | -0.41 | 11394 | 55.23 | 0.07 | -0.42 | 1609 | 55.75 | 0.27 | -0.22 | 979 | 53.63 | 0.23 | -0.22 | 1549 | 58.81 | 0.15 | -0.19 | 1095 | 58.08 | -0.03 | -0.34 |
| *Geococcyx californianus* | EU410488 | 17091 | 55.89 | 0.16 | -0.44 | 11395 | 55.12 | 0.10 | -0.44 | 1588 | 57.24 | 0.22 | -0.18 | 984 | 52.44 | 0.22 | -0.21 | 1541 | 57.88 | 0.15 | -0.21 | / | / | / | / |
| *Centropus bengalensis* | MN356332 | 17107 | 59.91 | 0.10 | -0.35 | 11400 | 59.57 | 0.04 | -0.36 | 1592 | 58.61 | 0.21 | -0.16 | 983 | 57.58 | 0.21 | -0.17 | 1546 | 61.13 | 0.13 | -0.17 | 1328 | 62.73 | -0.03 | -0.30 |
| *Centropus sinensis* | KT947122 | 17159 | 59.44 | 0.11 | -0.37 | 11397 | 59.42 | 0.06 | -0.38 | 1587 | 57.84 | 0.21 | -0.18 | 985 | 56.04 | 0.24 | -0.18 | 1543 | 59.95 | 0.15 | -0.18 | 216 | 64.35 | 0.27 | -0.77 |
| *Centropus unirufus* | NC052811 | 17089 | 58.67 | 0.12 | -0.37 | 11400 | 58.26 | 0.05 | -0.37 | 1594 | 57.53 | 0.23 | -0.20 | 977 | 56.09 | 0.21 | -0.15 | 1547 | 60.31 | 0.14 | -0.18 | / | / | / | / |

**Table S2** AT/GC skews in the mitochondrial protein-coding genes (PCG), 2 rRNA genes and the entire mitogenomes of Cuculidae birds.

| AT-skew = A−T/A+T | | | | | | | | | | | | | | | |
|---|---|---|---|---|---|---|---|---|---|---|---|---|---|---|---|
| Gene | 1 | 2 | 3 | 4 | 5 | 6 | 7 | 8 | 9 | 10 | 11 | 12 | 13 | 14 | 15 |
| *ND1* | 0.04 | 0.06 | 0.03 | 0.03 | 0.05 | 0.02 | 0.05 | 0.02 | 0.02 | 0.03 | 0.09 | 0.02 | **-0.01** | 0.01 | 0.03 |
| *ND2* | 0.18 | 0.22 | 0.18 | 0.18 | 0.13 | 0.19 | 0.16 | 0.20 | 0.20 | 0.16 | 0.11 | 0.16 | 0.14 | 0.15 | 0.16 |
| *ND3* | 0.13 | 0.08 | 0.10 | 0.08 | 0.12 | 0.07 | 0.05 | 0.07 | 0.07 | 0.11 | 0.06 | 0.06 | 0.02 | 0.01 | **-0.01** |
| *ND4* | 0.17 | 0.23 | 0.16 | 0.15 | 0.16 | 0.17 | 0.13 | 0.17 | 0.17 | 0.16 | 0.12 | 0.15 | 0.09 | 0.10 | 0.11 |
| *ND4L* | 0.10 | 0.16 | 0.11 | 0.10 | 0.07 | 0.14 | 0.09 | 0.11 | 0.11 | 0.10 | 0.12 | 0.11 | 0.03 | 0.07 | 0.01 |
| *ND5* | 0.20 | 0.25 | 0.19 | 0.18 | 0.17 | 0.20 | 0.19 | 0.21 | 0.20 | 0.18 | 0.17 | 0.20 | 0.11 | 0.16 | 0.14 |
| *ND6* | 0.58 | **-0.60** | **-0.59** | **-0.59** | **-0.54** | **-0.55** | **-0.53** | **-0.60** | **-0.55** | **-0.56** | **-0.56** | **-0.60** | **-0.52** | **-0.51** | **-0.53** |
| *COXI* | 0.08 | 0.09 | 0.06 | 0.06 | 0.04 | 0.09 | 0.05 | 0.08 | 0.08 | 0.06 | 0.04 | 0.06 | 0.00 | 0.01 | 0.01 |
| *COXII* | 0.11 | 0.16 | 0.16 | 0.14 | 0.11 | 0.15 | 0.10 | 0.13 | 0.13 | 0.10 | 0.16 | 0.18 | 0.10 | 0.12 | 0.10 |
| *COXIII* | 0.09 | 0.12 | 0.10 | 0.09 | 0.09 | 0.10 | 0.09 | 0.11 | 0.11 | 0.07 | 0.11 | 0.10 | 0.03 | 0.04 | 0.05 |
| *ATP6* | 0.10 | 0.13 | 0.08 | 0.05 | 0.07 | 0.13 | 0.07 | 0.09 | 0.09 | 0.14 | 0.11 | 0.14 | 0.08 | 0.10 | 0.10 |
| *ATP8* | 0.25 | 0.22 | 0.17 | 0.17 | 0.22 | 0.27 | 0.30 | 0.26 | 0.27 | 0.15 | 0.25 | 0.22 | 0.12 | 0.09 | 0.03 |
| *Cytb* | 0.10 | 0.15 | 0.10 | 0.08 | 0.08 | 0.10 | 0.09 | 0.09 | 0.08 | 0.08 | 0.04 | 0.12 | 0.01 | 0.05 | 0.06 |
| *12S RNA* | 0.24 | 0.21 | 0.19 | 0.20 | 0.16 | 0.20 | 0.18 | 0.21 | 0.21 | 0.21 | 0.23 | 0.22 | 0.21 | 0.24 | 0.21 |
| *16S rRNA* | 0.23 | 0.25 | 0.22 | 0.23 | 0.22 | 0.22 | 0.21 | 0.22 | 0.21 | 0.19 | 0.27 | 0.22 | 0.21 | 0.21 | 0.23 |
| 13PCG | 0.15 | 0.13 | 0.09 | 0.09 | 0.08 | 0.10 | 0.08 | 0.10 | 0.10 | 0.08 | 0.07 | 0.10 | 0.04 | 0.06 | 0.05 |
| overall | 0.16 | 0.18 | 0.18 | 0.15 | 0.14 | 0.16 | 0.14 | 0.16 | 0.15 | 0.14 | 0.14 | 0.16 | 0.10 | 0.11 | 0.12 |

| GC-skew =G−C/G+C | | | | | | | | | | | | | | | |
|---|---|---|---|---|---|---|---|---|---|---|---|---|---|---|---|
| Gene | 1 | 2 | 3 | 4 | 5 | 6 | 7 | 8 | 9 | 10 | 11 | 12 | 13 | 14 | 15 |
| *ND1* | -0.45 | -0.44 | -0.42 | -0.43 | -0.44 | -0.42 | -0.42 | -0.41 | -0.41 | -0.41 | -0.48 | -0.45 | -0.40 | -0.40 | -0.43 |
| *ND2* | -0.56 | -0.58 | -0.54 | -0.54 | -0.50 | -0.57 | -0.54 | -0.57 | -0.59 | -0.58 | -0.54 | -0.61 | -0.54 | -0.56 | -0.54 |
| *ND3* | -0.54 | -0.46 | -0.49 | -0.47 | -0.49 | -0.44 | -0.48 | -0.44 | -0.44 | -0.51 | -0.50 | -0.53 | -0.45 | -0.45 | -0.41 |
| *ND4* | -0.55 | -0.58 | -0.55 | -0.55 | -0.53 | -0.55 | -0.54 | -0.55 | -0.55 | -0.54 | -0.54 | -0.58 | -0.50 | -0.50 | -0.51 |
| *ND4L* | -0.50 | -0.49 | -0.46 | -0.44 | -0.44 | -0.50 | -0.45 | -0.46 | -0.48 | -0.49 | -0.48 | -0.54 | -0.35 | -0.41 | -0.35 |
| *ND5* | -0.51 | -0.52 | -0.51 | -0.51 | -0.50 | -0.49 | -0.50 | -0.49 | -0.48 | -0.51 | -0.51 | -0.54 | -0.43 | -0.46 | -0.47 |
| *ND6* | -0.65 | **0.65** | **0.63** | **0.62** | **0.62** | **0.63** | **0.62** | **0.61** | **0.63** | **0.64** | **0.62** | **0.68** | **0.58** | **0.61** | **0.59** |
| *COXI* | -0.32 | -0.31 | -0.28 | -0.29 | -0.29 | -0.30 | -0.29 | -0.30 | -0.30 | -0.29 | -0.30 | -0.34 | -0.27 | -0.28 | -0.26 |
| *COXII* | -0.35 | -0.35 | -0.35 | -0.36 | -0.36 | -0.34 | -0.31 | -0.34 | -0.33 | -0.35 | -0.37 | -0.38 | -0.30 | -0.32 | -0.31 |
| *COXIII* | -0.38 | -0.35 | -0.35 | -0.35 | -0.35 | -0.35 | -0.34 | -0.37 | -0.36 | -0.36 | -0.37 | -0.37 | -0.32 | -0.32 | -0.31 |
| *ATP6* | -0.57 | -0.62 | -0.58 | -0.58 | -0.56 | -0.59 | -0.56 | -0.56 | -0.56 | -0.59 | -0.58 | -0.60 | -0.53 | -0.55 | -0.52 |
| *ATP8* | -0.80 | -0.77 | -0.80 | -0.81 | -0.74 | -0.77 | -0.82 | -0.71 | -0.73 | -0.69 | -0.80 | -0.75 | -0.74 | -0.71 | -0.69 |
| *Cytb* | -0.47 | -0.50 | -0.47 | -0.48 | -0.48 | -0.46 | -0.47 | -0.46 | -0.46 | -0.48 | -0.46 | -0.52 | -0.41 | -0.45 | -0.42 |
| *12S rRNA* | -0.15 | -0.15 | -0.15 | -0.15 | -0.14 | -0.15 | -0.14 | -0.16 | -0.15 | -0.15 | -0.22 | -0.21 | -0.17 | -0.18 | -0.15 |
| *16S rRNA* | -0.16 | -0.17 | -0.17 | -0.16 | -0.17 | -0.17 | -0.16 | -0.16 | -0.16 | -0.13 | -0.22 | -0.18 | -0.16 | -0.18 | -0.20 |
| 13PCG | -0.48 | -0.43 | -0.40 | -0.41 | -0.40 | -0.41 | -0.40 | -0.40 | -0.40 | -0.41 | -0.42 | -0.44 | -0.36 | -0.38 | -0.37 |
| overall | -0.41 | -0.42 | -0.41 | -0.39 | -0.39 | -0.40 | -0.39 | -0.40 | -0.40 | -0.39 | -0.41 | -0.44 | -0.35 | -0.37 | -0.37 |

Note: The number in the 1$^{st}$ row of this table represent the species number as Table 1.

**Table S3** Base composition and AT/GC skews for the 13 PCGs in the mitogenome of *C. coromandus*.

| Name | A(%) | T(%) | C(%) | G(%) | A+T(%) | C+G(%) | AT-skew | GC-skew |
|---|---|---|---|---|---|---|---|---|
| PCGs | 31.66 | 23.41 | 33.21 | 11.72 | 55.07 | 44.93 | 0.15 | -0.48 |
| 1st | 28.58 | 23.31 | 29.60 | 18.51 | 51.89 | 48.11 | 0.10 | -0.23 |
| 2nd | 25.52 | 33.26 | 30.50 | 10.72 | 58.78 | 41.22 | -0.13 | -0.48 |
| 3th | 40.87 | 13.67 | 39.53 | 5.93 | 54.54 | 45.46 | 0.50 | -0.74 |

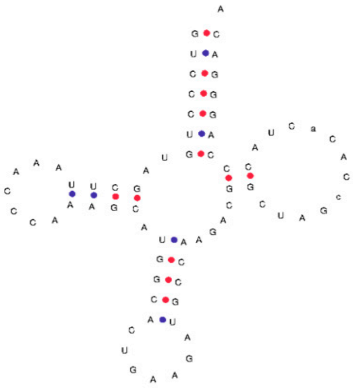

Phe(GAA)

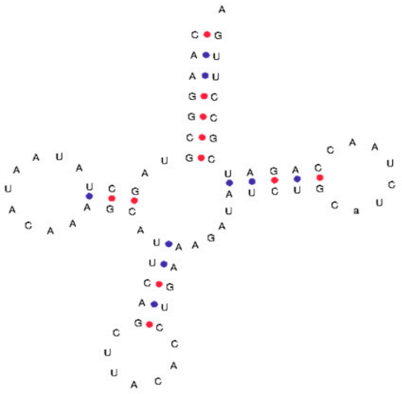

Val(TAC)

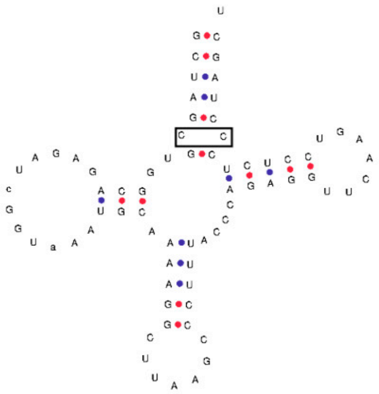

Leu(TAA)

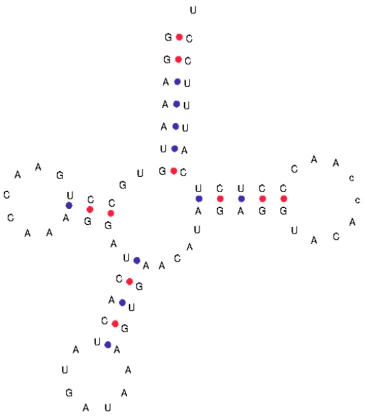

Ile(GAT)

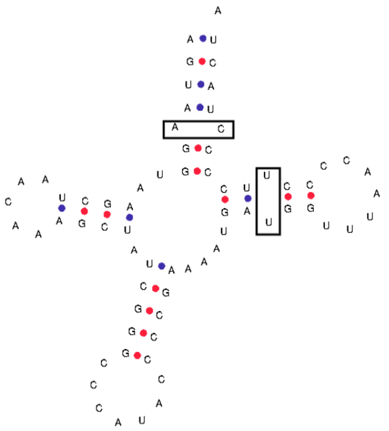

Met(CAT)

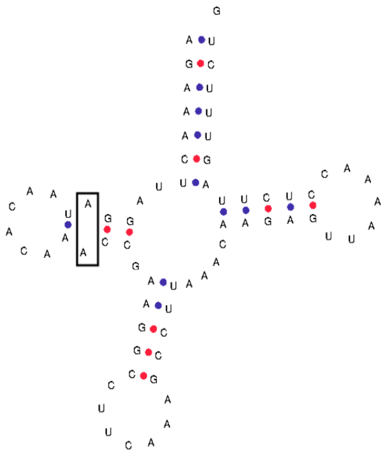

Trp(TCA)

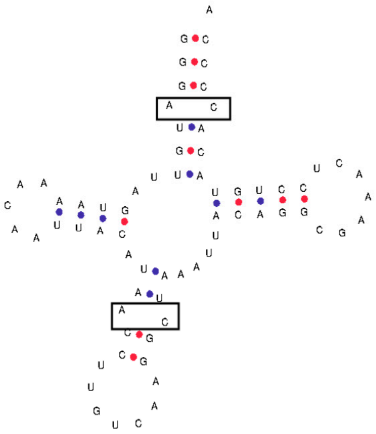

Asp(GTC)

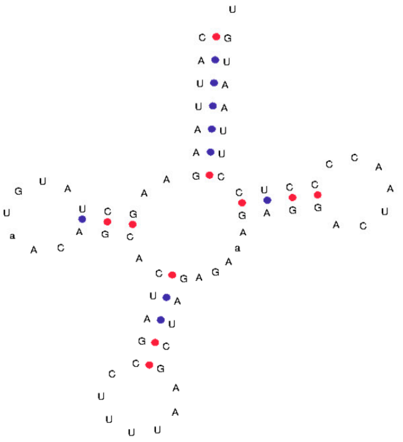

Lys(TTT)

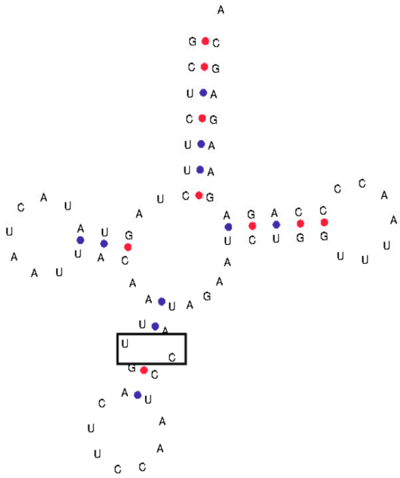

Gly(TCC)

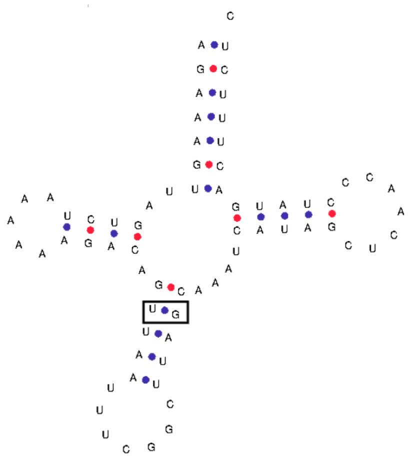

Arg(TCG)

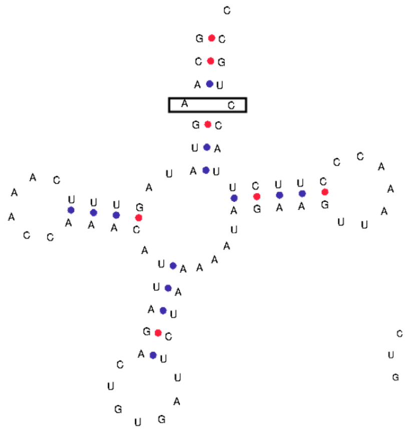

His(GTG)

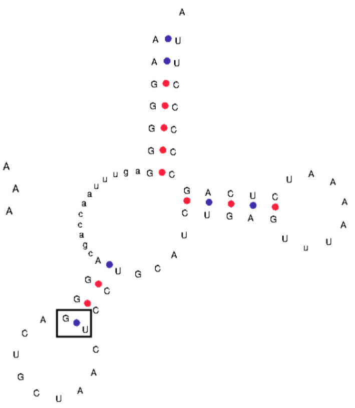

Ser(GCT)

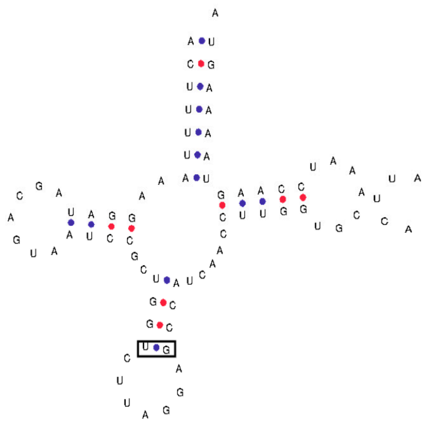

Leu(TAG)

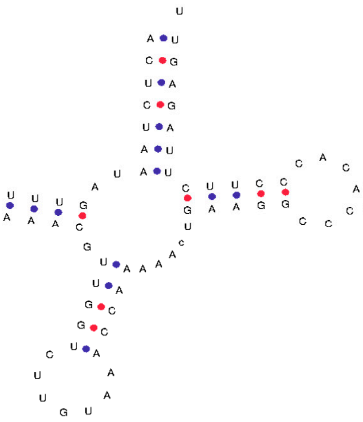

Thr(TGT)

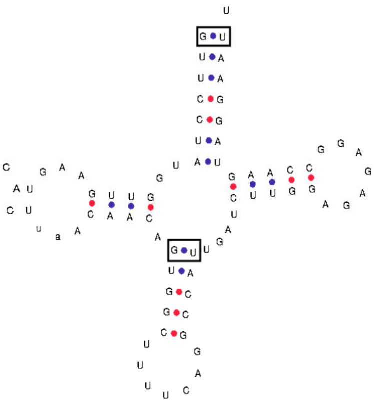

Gllu(TTC)

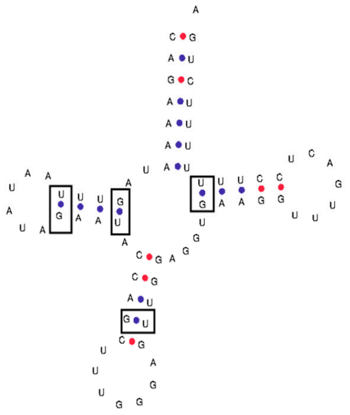

Pro(TGG)

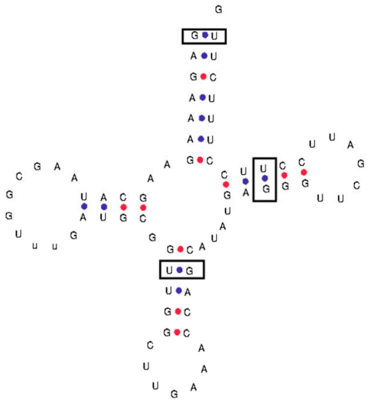

Ser(TGA)

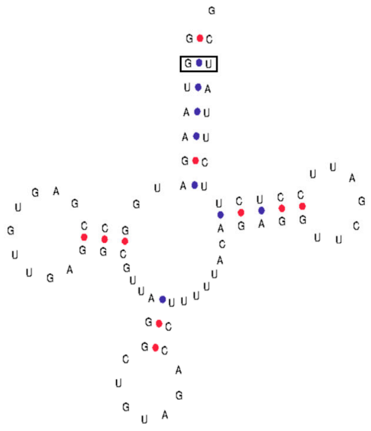

Tyr(GTA)

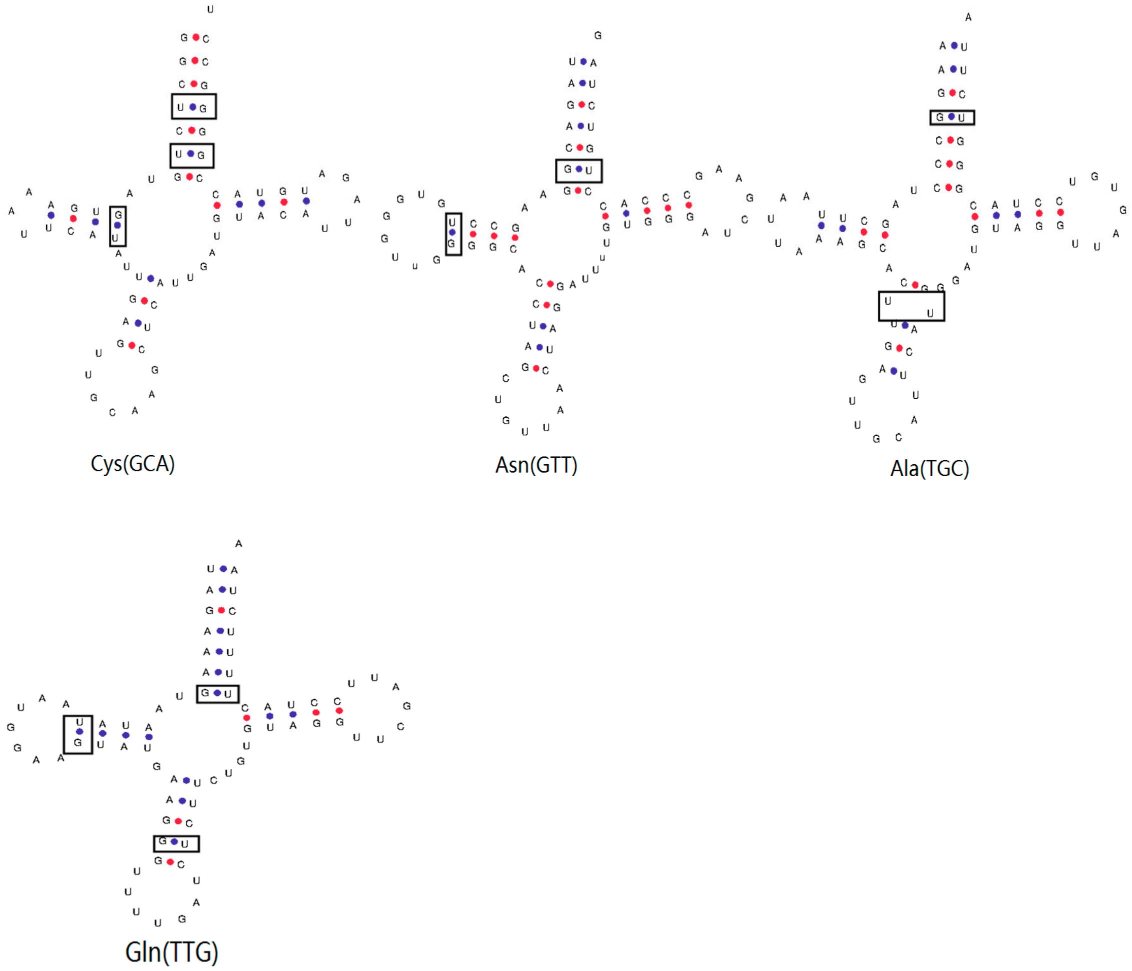


Figure. S1 Putative tRNA secondary structures predicted from the 22 tRNA gene sequences found in the mitogenome of *C. coromandus*. The black box in the figure is the mismatched position.

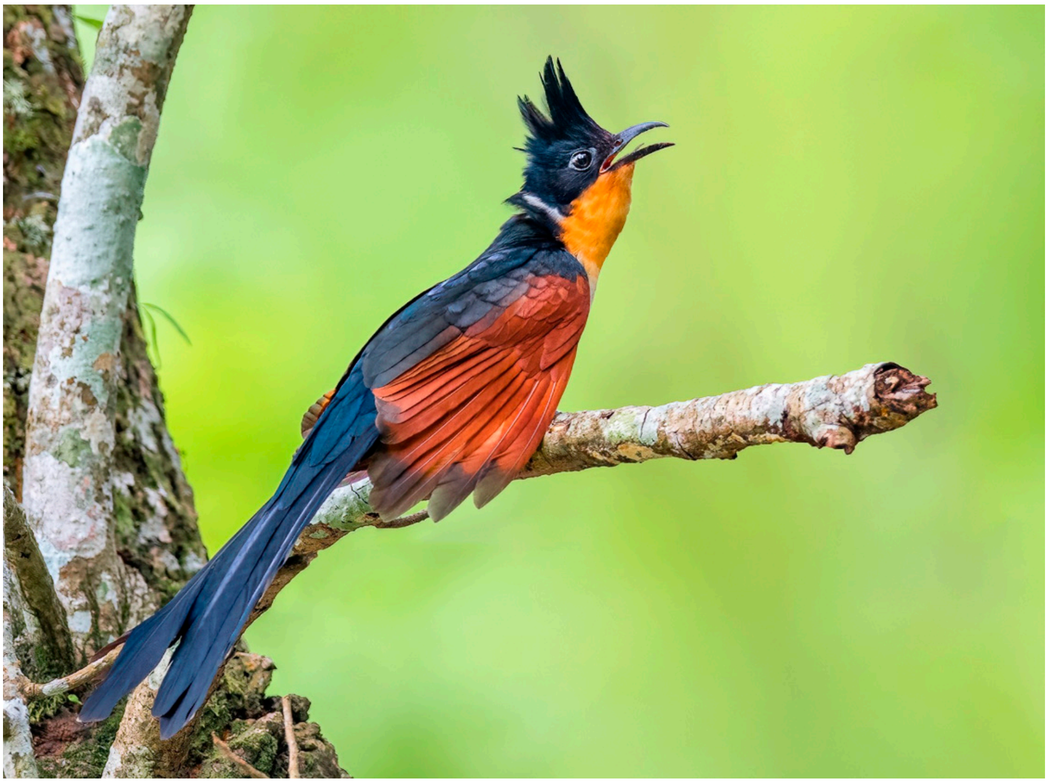

Figure S2 Photograph of *C. coromandus* (Chestnut-winged Cuckoo - *Clamator coromandus* - Birds of the World https://birdsoftheworld.org/bow/species/chwcuc1/cur/introduction).
